# Maternal mortality and its prominence in the Syrian Arab Republic: Challenges, efforts, and recommendations

**DOI:** 10.1016/j.amsu.2022.104584

**Published:** 2022-09-08

**Authors:** Malaika Saeed Butt, Zoaib Habib Tharwani, Muhammad Muzzamil, Hania Mansoor Rafi

**Affiliations:** Dow Medical College, Dow University of Health Sciences (DUHS), Karachi, Pakistan; Health Services Academy, Islamabad, Pakistan; Dow Medical College, Dow University of Health Sciences (DUHS), Karachi, Pakistan

**Keywords:** MMR, Syria, Maternal mortality, Maternal mortality rate, Maternal mortality ratio, COVID-19 pandemic, Syrian Arab Republic

## Abstract

WHO has brought to light how the Maternal Mortality Ratio is alarmingly high in countries like Syria because women lack basic necessities such as access to proper healthcare, resources, and information. With the ongoing war, COVID-19 pandemic, and other resultant factors are converging to further exacerbate Syria's current collapsing situation. Leading to an increasing number of maternal mortality cases, with the country's economy and its disparities making it nearly impossible for Syria to recover. In the wake of these problems piling up, Syria requires immediate preventative measure to be put into place to avoid further crises complications, and mortalities.

## 1 Introduction

Maternal mortality, according to the World Health Organization (WHO), is the death of a female as a result of pregnancy-related complications [1]. The maternal mortality ratio (MMR) measures maternal deaths per 100,000 live births (excluding unintentional or incidental causes) during a specified (42 day) time period [2].

The United Nations (UN) estimates that 350,000 women die due to such complications each year, with 99% of these maternal fatalities occurring in third-world nations [3]. Even though globally, the MMR fell from 342/100,000, in 2000, to 211/100,000, in 2017, in low-income war zone nations (i.e.: Syria, Afghanistan, Yemen), these rates still remain relatively high [4].

The United Nations Children's Fund (UNICEF) reported the MMR in Syria was estimated at 31 deaths per 100,000 livebirths in 2017 [5]. Various factors over the years have influenced the MMR in Syria, of which the long going civil war is a major one. This is shown by the fact that before the civil war in Syria begun, the MMR was 52/100,000 livebirths in 2009, and during the peak of the war, in 2015, it was reported to be at 68/100,000 livebirths [6]. Additionally, the lack of proper healthcare, shortage of resources, waves of human displacement, and multiple infectious illnesses have influenced the MMR for the words [[7], [8], [9]]. On top of all of the aforementioned factors, the COVID-19 pandemic went onto exacerbate the already on edge healthcare system-wide collapse making it more difficult to control the overall situation [10].

This commentary focuses in on the Maternal Mortality in Syria, the multiple variables contributing to its prominence in the country, and recommendations on how to tackle the underlying problems using a multi-faceted approach.

## 2 Challenges

Maternal mortality still remains a great concern in many countries, especially in the developing world. The Syrian Arab Republic, being one of the developing countries, also struggles with this issue. The civil war in Syria started in 2011, and led to multiple crises in the country, one of which included destruction of multiple medical services in the country [6]. This is shown by WHO HeRAMS (Health Resources and Services Availability Monitoring System) report, which states that out of the 113 assessed public hospitals only 50% were reported to be fully functioning, leaving significantly less facilities for the gynecological sector, therefore contributing to the barrier in reducing MMR [6].

Moreover, the Assad regime along with the Russian alliance targeted the healthcare workers (HCWs) by persecution, arrest, torture, and killings [11]. This is shown in a report by Physicians for Human Rights (PHR) in June 2022, which says that the Syrian government and their Russian allies were responsible for 93% of the deaths of medical personnel [11]. This creates a fear in the minds of healthcare professionals due to which around 70% fled the country contributing to the lack of HCWs and thus high MMR [12].

To improve the healthcare situation in any country, it is imperative to have the government's support. In Syria however, various factors are limiting the government's aid, which imposes yet another barrier against the efforts to reduce MMR. A major obstacle affecting the government was the oppression of Syrian-Russian military alliance which targeted the civilians and their households in 2020. Due to this, over 50% of the healthcare facilities were destroyed, leaving the nation underprepared for the pandemic at the time [13]. Because of these instabilities, major humanitarian funding by the UN was also frozen, resulting in a severe medical equipment and supplies shortage, further crippling the healthcare system [13].

The advent of COVID-19 in Syria was another major factor which affected the MMR. As of August 25th^,^ 2022, 55,924 COVID-19 infections and 3,162 COVID related deaths have been reported in Syria, and around 4.2 million COVID vaccine doses have been administered, which is enough for only 12.4% of the population [14,15]. COVID-19, along with the ongoing war, had a huge impact on the country's economy due to which inflation reached all-time high as the price of basic food items went up by 236% and the Syrian currency lost 82% of its value against the dollar [16]. This left the citizens living below the poverty line (90% Syrians) deprived of proper healthcare facilities, therefore affecting the MMR for the worse [17]. Moreover, in the present year (2022) food insecurity rose up due to COVID, affecting over 80% of the population [17]. These alarming rates of food insecurity prove to be harmful for the pregnant women, increasing the probability of a rise in MMR [18].

Considering all the above-mentioned reasons, it is safe to assume that all these factors add up to affect the surveillance of MMR in Syria, which is proven by the fact that the recent numbers of MMR available are from 5 years ago, i.e. 2017 [19]. Therefore, even though the numbers may appear to be modest compared to other countries, in reality they could be significantly high.

In the long run, all of this could be significantly detrimental as high rates of maternal mortality are also associated with an ever-increasing risk of child mortality, putting the future of Syria into jeopardy [20].

## 3 Efforts

Various non-governmental organizations (NGOs) and UN agencies, organized a humanitarian response to cater to the consequent humanitarian need in Syria [21].

A mission led by the United Nations Population Fund (UNFPA), a sexual and reproductive health agency, in Tal Refaat and surrounding areas of Northern Syria, focused on the introduction and implementation of three mobile clinics: one operated by the Syrian Arab Red Crescent as a general health clinic while two run by Mother Saint James the Mutilated (MSJM) as sexual and reproductive health clinics [22]. They also carried out the distribution of sanitary and hygiene essentials, as well as winter protection kits for women [22]. In north-west Syria WHO assisted 9 hospitals in resuming operations as well as in the restoration of 3 primary healthcare centers in Aleppo and rural Damascus in 2018 [23]. Such campaigns funded by local and international organizations are an effective way of enabling direct and easy access to basic health facilities and supplies to women regardless of the geographical location or socioeconomic standing, along with the provision of reproductive health education and fostering healthy and hygienic practices.

The systematic targeting of health care facilities and healthcare personnel since the emergence of the crisis, particularly in North West Syria, has fractured and fragmented the Syrian health sector, leaving pregnant women and girls with limited alternatives [21,24]. In light of this, an initiative for the protection of HCWs and infrastructure in North West Syria was launched by Geneva Call in collaboration with IHH Humanitarian Relief Foundation. Through distribution and exhibition of posters in medical facilities like hospitals, dispensaries, and pharmacies, and social media campaigns, this initiative aimed at promoting healthcare protection in the Syrian conflict [24].

## 4 Recommendations

Restoring Syria's healthcare system is challenging, however investments made towards the development of contemporary approaches such as field hospitals, mobile clinics and emergency obstetric care facilities can help humanitarian organizations expand their reach and aid in the deployment of medical services to inaccessible regions, especially for women afflicted by the crisis [21]. The targeted health facilities that have been reconfigured as field hospitals to cater the medical and humanitarian requirements of the impacted communities are also met with insufficient medical equipment and trained healthcare professionals [25]. Therefore, alongside setting up medical camps for the provision of medical services and supplies to impacted communities, the United Nations and other humanitarian organizations should devise programs for the training of medical personnel which is imperative to overcoming the persistent deficit of staff in healthcare facilities. Despite the institution of the Geneva Conventions and the International Human Law, medical facilities and medical personnel remain under the threat of violence and continue to be weaponized [25]. The gravity of the implications of targeting healthcare facilities and workers must be addressed in light of the International Humanitarian Law and the Syrian government should be held accountable for its violation [26].

Despite the worsening of all major health indicators, conflict-affected regions reportedly obtain an average of 60% less funding for reproductive healthcare [27]. The sanctions imposed on Syria have proven to be more detrimental for the civilians than the Syrian government and a sheer volume of the humanitarian aid provided to Syria by western countries is received either by refugees fleeing the nation or parts of Syria seized by militant groups acting against the Syrian government, leaving the Government controlled areas, comprising 70% of the country, unaided [28]. It is crucial for the international community and humanitarian relief organizations to negotiate with the Syrian government and to map out focused schemes for redirecting their services and funding towards conflict-affected areas in need of aid. By doing so, an improvement may be observed in the basic healthcare as well as antenatal and postnatal care services reaching women residing in these areas, which may in turn help ameliorate the MMR. Although significant efforts have been made in promoting the participation of women in governance and decision making, female representation within this sphere remains inadequate [29]. This becomes a barrier in bringing to the forefront issues faced by women in real time and downplays the severity of the circumstances [29]. Moreover, women in need of legal and psychological assistance are unable to access the concerned bodies making their survival difficult. Campaigns and negotiations spearheaded by female leaders are a requisite for addressing the needs of women who are affected by armed conflict, including demands for appropriate reproductive health services and life-saving interventions which would be effective in averting a large percentage of maternal deaths and disabilities [29,30].

Additionally, the COVID-19 pandemic brought to surface the problems of the healthcare leadership in Syria and corroborated the need for flexible, skilled and gender-inclusive healthcare leadership [31]. In order to fill this gap and reinforce Syria's healthcare leadership through capable personnel, regional or international institutions with established medical and healthcare leadership programs should formulate apposite curricula for the medical personnel that would equip them with the needed expertise [31].

## 5 Conclusion

In conclusion, despite the underlying circumstances plaguing the maternal population in Syria, swift and drastic action by both public and private organizations as well as policy-making bodies may render a substantive change. It is absolutely imperative for both the economic and holistic health of women in Syria that the underlying humanitarian crisis be solved first. Measures such as increased female representation, increased funding for provisional health facilities, introduction of context-relevant programs of medical personnel and implementation of systemic healthcare systems, is a first step towards controlling the increasing rates of maternal mortality in Syria averting further critical complications.

## Ethical approval

N/A.

## Sources of funding

None.

## Author contribution

All authors equally contributed.

## Registration of research studies

Name of the registry: N/A.

Unique Identifying number or registration ID: N/A.

Hyperlink to your specific registration (must be publicly accessible and will be checked): N/A.

## Guarantor

N/A.

## Consent for publication

All authors agreed to the publication of this manuscript.

## Provenance and peer review

Not commissioned, externally peer reviewed.

## Declaration of competing interest

None declared.
